# Supplementary material for: Hypoxia-Regulated CD44 and xCT Expression Contributes to Late Postoperative Epilepsy in Glioblastoma
Source: Biomedicines. 2025 Feb 5;13(2):372. doi: 10.3390/biomedicines13020372 (PMC11853413; doi:10.3390/biomedicines13020372)
Supplement: Supplementary file 1 [file biomedicines-13-00372-s001.zip › Supplementary Figure legends proof reading.pdf]

Supplementary Figure legends

Supplementary Figure S1. **a)** Classification of GBM phenotypes based on MRI. GBM is divided into high-invasive and low-invasive phenotypes based on characteristic findings of Gd-enhanced T1WI and FLAIR on MRI. Upper pictures show high-invasive GBM, displaying an irregular margin with heterogeneous enhancement. FLAIR image depicts diffusely extended peritumoral brain edema. Lower pictures show low-invasive GBM, with a relatively demarcated tumor margin with intensely, homogeneously enhanced tumor margin. FLAIR image shows relatively localized peritumoral brain edema. **b)** Illustration showing the operative method of sampling tumor tissues from the core and periphery in GBM using an image-guided neuro-navigation system with assistance of the fence post technique.

Supplementary Figure S2. Treatment of GSCs with SSZ promoted the cleaved poly-ADP ribose polymerase (PARP) in 5% O_2 hypoxic condition, demonstrating induction of apoptosis in all GSCs.

Supplementary Figure S3. Effects of hypoxia (1% and 5% O_2) on expressions of CD44, xCT, and EAAT2 in the three GSC lines. A tendency toward severe hypoxia increasing CD44 and EAAT2 expression and decreasing xCT expression was seen in all GSC lines compared to moderate hypoxia. Changes of CD44 and xCT expression between the two hypoxias were significant only in GSC-2.

Supplementary Figure S4. Features of the 7 patients who developed epilepsy after tumor resection among the 23 patients with GBM. **a)** Bar graphs showing duration times “from surgery to occurrence of epilepsy”, “from surgery to detection of tumor recurrence (progression-free survival time)”, “from occurrence of epilepsy to detection of tumor recurrence”, and “overall survival (OS) time”. All patients developing epileptic attack presented attacks one month after surgery and before detection of tumor recurrence on imaging including MRI and methionine positron emission tomography (Met-PET). **b)** Kaplan-Maier survival curves of GBM patients with late epilepsy. Patients who had a duration from occurrence of epilepsy to recurrence longer than 2.5 months (L group, four patients: Cases 1, 2, 4, and 5) achieved much longer survival than those with a duration up to 2.5 months (S group, three patients: Cases 3, 6, and 7), although the difference between groups was not significant. M, months; NA, not assessed.

Supplementary Figure S5. Bar graphs showing oxygen consumption rate in hypoxic conditions in the three GSC lines evaluated by an XFp extracellular flux analyzer. Right: basal respiration.

Left: ATP production. All three GSC lines show increases in both respiration rate and ATP production under 5% hypoxia compared to 1%, indicating more active proliferation of GSCs under 5% hypoxia. ns, not significant.
