# Supplementary material for: Hypoxia-Regulated CD44 and xCT Expression Contributes to Late Postoperative Epilepsy in Glioblastoma
Source: Biomedicines. 2025 Feb 5;13(2):372. doi: 10.3390/biomedicines13020372 (PMC11853413; doi:10.3390/biomedicines13020372)
Supplement: Supplementary file 1 [file biomedicines-13-00372-s001.zip › Supplementary materials.pdf]

Contents of Stem Cell Medium: Medium comprised 10 µg/ml insulin (Wako, Osaka, Japan), 10 nmol/l recombinant human basic fibroblast growth factor, 10 nmol/l recombinant human epidermal growth factor, 5 µmol/l heparin, N2 supplement (Wako), GlutaMAX supplement (GIBCO, Waltham, Massachusetts, the united state of America (USA)), and penicillin/streptomycin/amphotericin B mixture (neural stem cell medium). Growth factors were purchased from Pepro Tech (London, UK).

Procedure for CD44-Knockdown: Lentiviral particles were generated using the shRNA expression vector pLKO.1-puro, which carries a shRNA sequence against CD44 (CD44 MISSION shRNA, SHCLNG-NM_000610; Sigma Aldrich, Saint Louis, Missouri, USA) together with the MISSION lentiviral packaging mix (SHP001; Sigma Aldrich), according to the instructions from the manufacturer. HEK 293T cells were co-transfected with the products generated in lentiviral particles which carry the shRNA sequence against CD44 using Lipofectamine 3000 reagent (Invitrogen, Waltham, Massachusetts, USA). The supernatant containing virus particles was harvested 48 hours after transfection and used to infect the GSCs. After 48 hours of incubation, infected cells were selected using puromycin (0.5 µg/ml; Invitrogen).
