# Supplementary figures and images for: Hypoxia-Regulated CD44 and xCT Expression Contributes to Late Postoperative Epilepsy in Glioblastoma
Source: Biomedicines. 2025 Feb 5;13(2):372. doi: 10.3390/biomedicines13020372 (PMC11853413; doi:10.3390/biomedicines13020372)

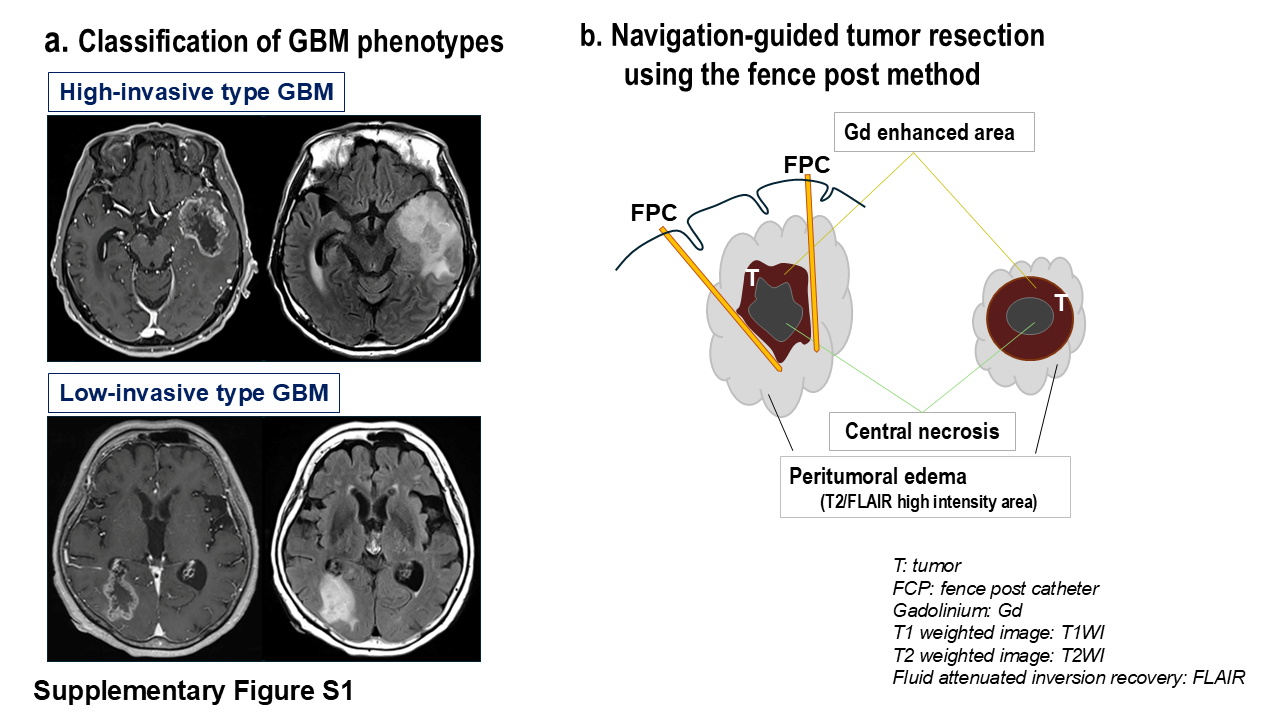

Supplement: Supplementary file 1 [file biomedicines-13-00372-s001.zip › Supplementary Figure S1.TIF]

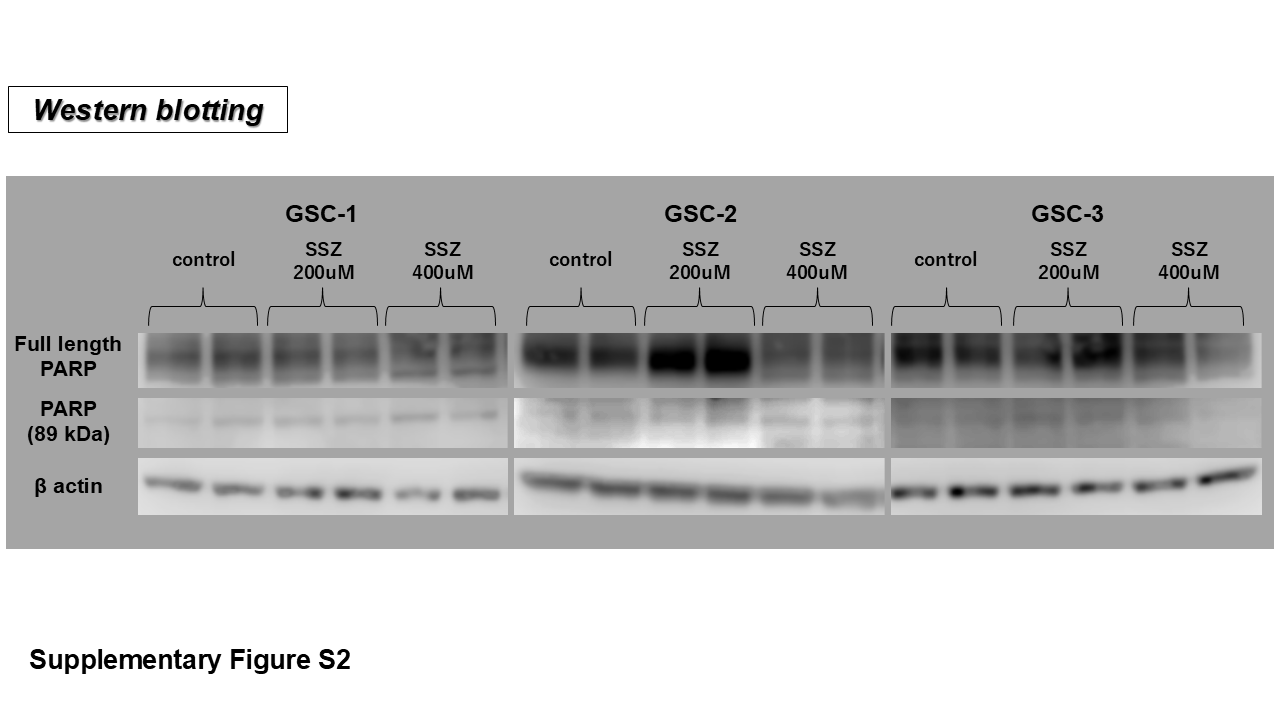

Supplement: Supplementary file 1 [file biomedicines-13-00372-s001.zip › Supplementary Figure S2.TIF]

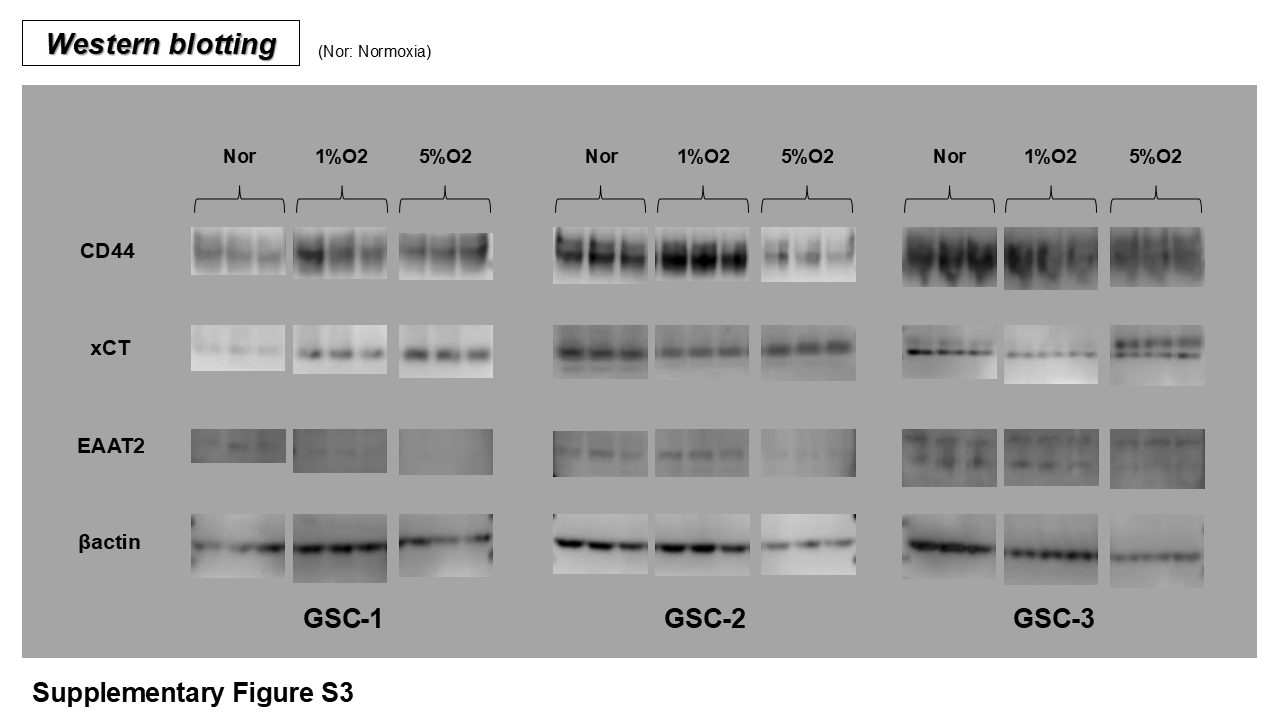

Supplement: Supplementary file 1 [file biomedicines-13-00372-s001.zip › Supplementary Figure S3.TIF]

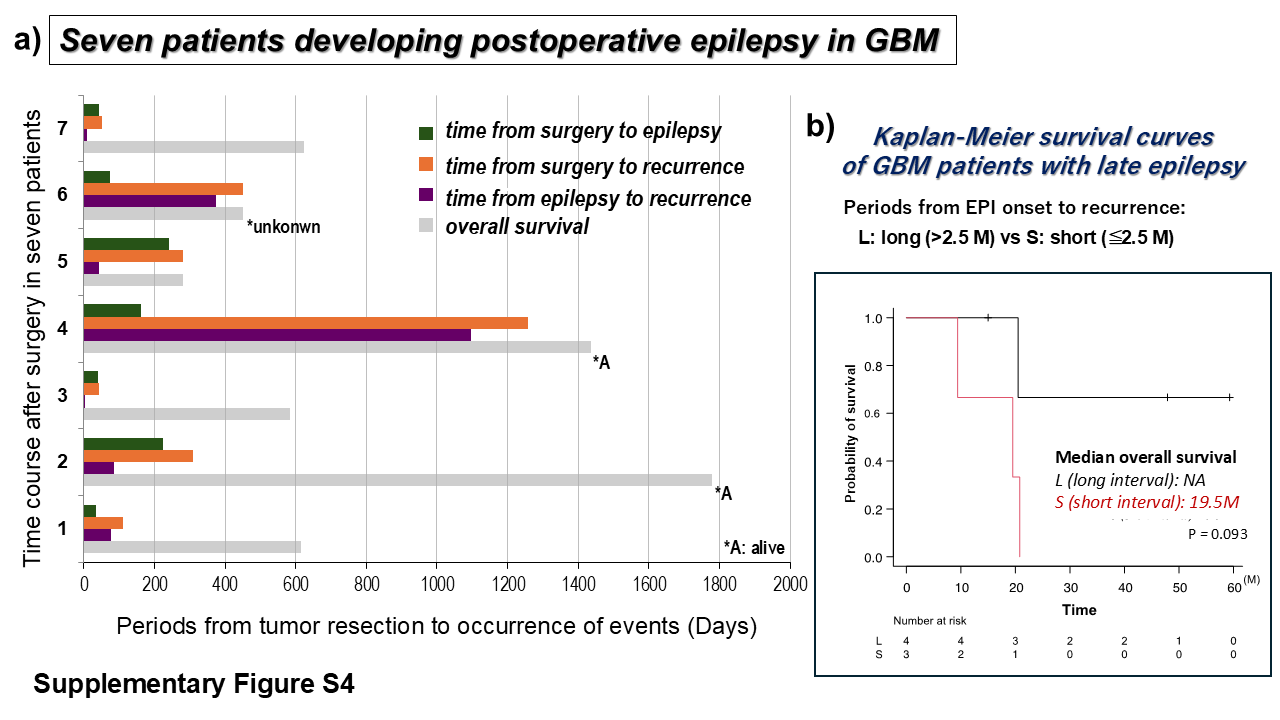

Supplement: Supplementary file 1 [file biomedicines-13-00372-s001.zip › Supplementary Figure S4.TIF]

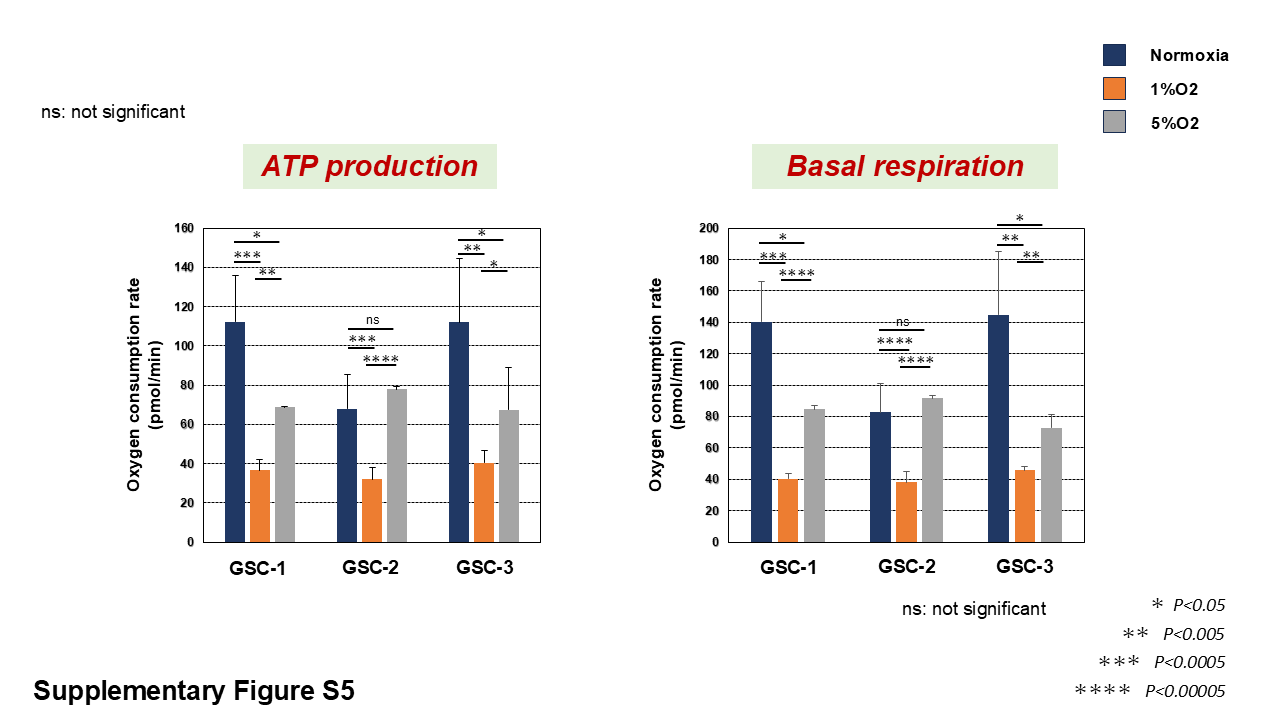

Supplement: Supplementary file 1 [file biomedicines-13-00372-s001.zip › Supplementary Figure S5.TIF]
